# Multi-transcriptome analysis following an acute skeletal muscle growth stimulus yields tools for discerning global and MYC regulatory networks

**DOI:** 10.1016/j.jbc.2022.102515

**Published:** 2022-09-21

**Authors:** Kevin A. Murach, Zhengye Liu, Baptiste Jude, Vandre C. Figueiredo, Yuan Wen, Sabin Khadgi, Seongkyun Lim, Francielly Morena da Silva, Nicholas P. Greene, Johanna T. Lanner, John J. McCarthy, Ivan J. Vechetti, Ferdinand von Walden

**Affiliations:** 1 Department of Health, Human Performance, and Recreation, Exercise Science Research Center, University of Arkansas, Fayetteville, Arkansas, USA; 2 Cell and Molecular Biology Graduate Program, University of Arkansas, Fayetteville, Arkansas, USA; 3 Department of Physiology and Pharmacology, Karolinska Institute, Solna, Sweden; 4 Department of Women’s and Children’s Health, Karolinska Institute, Solna, Sweden; 5 Center for Muscle Biology, University of Kentucky, Lexington, Kentucky, USA; 6 Department of Physical Therapy, University of Kentucky, Lexington, Kentucky, USA; 7 Cachexia Research Laboratory, University of Arkansas, Fayetteville, Arkansas, USA; 8 Department of Physiology, University of Kentucky, Lexington, Kentucky, USA; 9 Department of Nutrition and Health Sciences, University of Nebraska-Lincoln, Nebraska, USA

**Keywords:** myosin, gene transcription, muscle hypertrophy, Warburg effect, transcriptomics, 5-Ethenyl uridine, *Ankrd1*, *Runx1*, *Rpl3*, myonuclei, ChIP-seq, chromatin immunoprecipitation sequencing, DEG, differentially expressed gene, ECM, extracellular matrix, EU, 5-Ethenyl uridine, FANS, fluorescent activated nuclear-sorting, Lisa, Landscape *In Silico* deletion analysis, RNA-seq, RNA-sequencing

## Abstract

*Myc* is a powerful transcription factor implicated in epigenetic reprogramming, cellular plasticity, and rapid growth as well as tumorigenesis. Cancer in skeletal muscle is extremely rare despite marked and sustained *Myc* induction during loading-induced hypertrophy. Here, we investigated global, actively transcribed, stable, and myonucleus-specific transcriptomes following an acute hypertrophic stimulus in mouse plantaris. With these datasets, we define global and *Myc*-specific dynamics at the onset of mechanical overload-induced muscle fiber growth. Data collation across analyses reveals an under-appreciated role for the muscle fiber in extracellular matrix remodeling during adaptation, along with the contribution of mRNA stability to epigenetic-related transcript levels in muscle. We also identify *Runx1* and *Ankrd1* (*Marp1*) as abundant myonucleus-enriched loading-induced genes. We observed that a strong induction of cell cycle regulators including *Myc* occurs with mechanical overload in myonuclei. Additionally, *in vivo Myc*-controlled gene expression in the plantaris was defined using a genetic muscle fiber-specific doxycycline-inducible *Myc*-overexpression model. We determined *Myc* is implicated in numerous aspects of gene expression during early-phase muscle fiber growth. Specifically, brief induction of *Myc* protein in muscle represses *Reverbα*, *Reverbβ*, and *Myh2* while increasing *Rpl3*, recapitulating gene expression in myonuclei during acute overload. Experimental, comparative, and *in silico* analyses place *Myc* at the center of a stable and actively transcribed, loading-responsive, muscle fiber–localized regulatory hub. Collectively, our experiments are a roadmap for understanding global and *Myc*-mediated transcriptional networks that regulate rapid remodeling in postmitotic cells. We provide open webtools for exploring the five RNA-seq datasets as a resource to the field.

*Myc* is a transcription factor known to drive cellular plasticity (1, 2), epigenetic reprogramming toward stemness as a Yamanaka factor (3, 4, 5, 6), and rapid growth *via* proliferative and nonproliferative mechanisms (7, 8, 9). A proto-oncogene that dimerizes with MAX and interacts and/or complexes with numerous other proteins, (10, 11) MYC protein is a “universal amplifier” and “supermanager” of transcription with intricate and multifaceted functionality (12, 13, 14). Evidence for a role of MYC in syncytial muscle fiber growth emerged 20 to 30 years ago (15, 16, 17). A decade ago, MYC was hypothesized to be a key aspect of skeletal muscle adaptation to exercise (18). We recently found that the promoter region of *Myc* was hypomethylated in myonuclei following short-term mechanical overload of the mouse plantaris muscle (19). MYC protein binds the ribosomal DNA promoter during muscle overload (20), consistent with its influence on ribosome biogenesis and protein synthesis in striated muscle (21, 22). We also report that MYC-associated areas of ribosomal DNA are differentially methylated in murine myonuclei and human muscle tissue after acute loading (23). Dysregulation of *Myc* results in tumor development and maintenance in mononuclear cells (8, 9). Even transiently elevated MYC can cause tumors in some cells (24); however, *Myc* transcript and protein (MYC) may be elevated for up to 2 weeks during continuous mechanical overload in mouse muscle without an overt deleterious effect (23, 25, 26, 27, 28). MYC protein abundance in human muscle after resistance training also associates with the magnitude of hypertrophic adaptation (29). The unique multinuclear, terminally differentiated postmitotic nature of muscle fibers likely explains how muscle is resistant to developing cancer (30, 31, 32, 33) and why sustained MYC is tolerated in this tissue. Although the role of MYC in proliferative cells is well studied, its function as a transcription factor in postmitotic myonuclei during muscle growth is incompletely defined.

In the current investigation, we generated four interrelated murine muscle RNA-sequencing (RNA-seq) datasets using a proven hypertrophic loading stimulus (34, 35) to understand muscle tissue and myonucleus-specific transcriptional dynamics at the onset of rapid muscle growth. We focused on the effects of *Myc* given its (1) established role in driving nonproliferative tissue growth and overall cellular plasticity in nonmuscle cell types (1, 7) and (2) proposed role in human (23, 29, 36) and rodent (17, 20, 22, 25, 26, 27, 28) loading-induced skeletal muscle hypertrophy. We then performed a genetically driven muscle fiber–specific *in vivo Myc* overexpression experiment along with *in silico* chromatin immunoprecipitation sequencing (ChIP-seq) analysis to provide focused insight on how *Myc* may contribute to global gene expression during rapid muscle remodeling.

## Results

**Figure 1:**
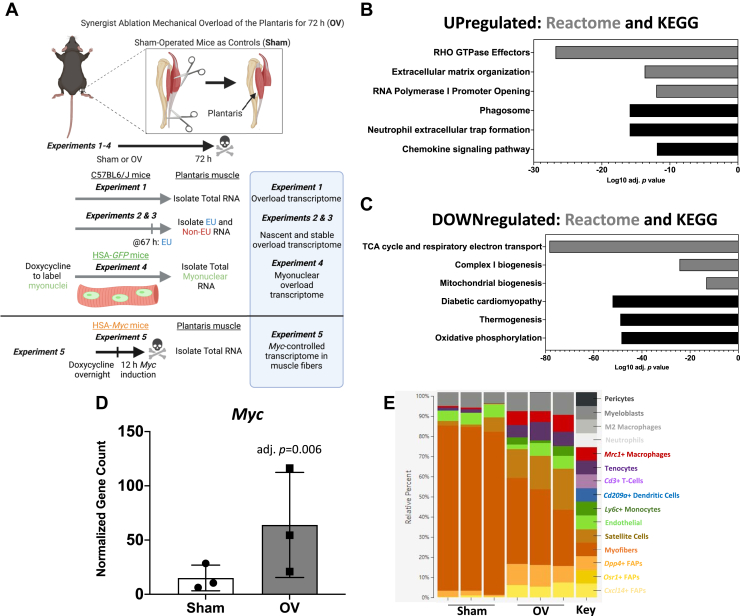
***Experiment 1*: RNA-sequencing of total RNA from plantaris muscle after 72 h of mechanical overload.***A*, study design schematic showing the conditions of *Experiments 1 to 5*. *B*, pathway analysis of upregulated genes in OV *versus* sham. *C*, pathway analysis of downregulated genes in OV *versus* sham. *D*, *Myc* mRNA levels in sham and overload determined by RNA-seq. *E*, digital deconvolution of muscle overload data using CIBERSORTx (42) and data from Oprescu *et al.* (43) to delineate cellular contributions to the global transcriptome. Normalized gene count, gene counts normalized using DESeq2. EU, 5-Ethenyl uridine; OV, overload.

Figure 1*A* is a study design schematic. *Experiment 1* defines the global transcriptome using RNA-seq after 72 h of synergist ablation mechanical overload of the mouse plantaris muscle. *Experiments 2 and 3* used the tissue from *Experiment 1* to provide information on the contribution of active transcription *versus* mRNA stability to global gene expression with overload. *Experiment 4* details the myonucleus-specific transcriptome during overload to identify muscle fiber–enriched genes, which is further informed by *Experiments 2* and *3*. Myonuclei only comprise ∼30% of all nuclei after short-term mechanical overload (19), so defining the transcriptome specifically in myonuclei is critical for understanding muscle fiber adaptation. The transcriptional regulation and localization of *Myc* and its impact on muscle gene expression were explored using data from *Experiments 1 to 4*. *Experiment 5* utilized a doxycycline-inducible muscle fiber–specific *in vivo Myc* pulse in the plantaris to understand what genes *Myc* controls within muscle fibers and how this relates to myonuclear gene expression during overload (*Experiment 4*); we corroborated the results from these analyses using computational ChIP-seq (37). All transcriptome data are publicly available for browsing:

*Experiments 1 to 3* https://liuzhengye.github.io/Hypertrophy/interactive_MA_PLOT_Eu.html

*Experiment 4* https://liuzhengye.github.io/Hypertrophy/interactive_MAplot_myonuclei.html

*Experiment 5* https://liuzhengye.github.io/Hypertrophy/interactive_MA_MYC.html

### Experiment 1: The global plantaris transcriptome after 72 h of mechanical overload

Pathway analysis of differentially regulated genes (false discovery rate adjusted *p* value [adj. *p*] < 0.05) in plantaris tissue after overload revealed extracellular matrix (ECM), inflammatory, histone (RNA Pol I), and RHO-GTPase gene expression were higher relative to sham (Fig. 1*B*) (Table S1 and S2). A large proportion of downregulated genes were related to oxidative metabolism (Fig. 1*C*) (Table S3). This repressed gene signature could contribute to a “Warburg effect” that occurs during rapid overload-induced muscle hypertrophy, marking a shift toward “aerobic glycolysis” for rapid biomass accumulation (38, 39, 40, 41). Consistent with our prior murine studies (19, 25, 26) and human resistance exercise time course data (23), *Myc* was higher after overload (Log2FC = 2.0, adj. *p* = 0.006) (Fig. 1*D*).

To provide insight on what cell types contribute to global gene expression profiles in sham and overload, we conducted digital deconvolution analysis with CIBERSORTx using *Experiment 1* transcriptome data (42). The analysis algorithm was trained using single cell RNA-seq data from a 10 days muscle regeneration dataset (43) (Fig. 1*E*). The interstitial cell proportion in muscle increases at the onset of overload, outnumbering myonuclei (19). Despite this shift, the largest contribution to gene expression in muscle was predicted to be from muscle fibers (*i.e.*, myonuclei) after 72 h of overload. The second largest contributions were from muscle stem cells (satellite cells) and fibro-adipogenic progenitors (Fig. 1*E*). We recently reported that successful ECM remodeling during the first 96 h of overload determines the long-term hypertrophic response (44). Early stage ECM remodeling is strongly influenced by satellite cells and fibro-adipogenic progenitors (44, 45); it follows that these cell types are major contributors to early-phase gene expression during growth.

### Experiments 2 and 3: Nascent and stable mRNA transcriptomes after 72 h of mechanical overload

**Figure 2:**
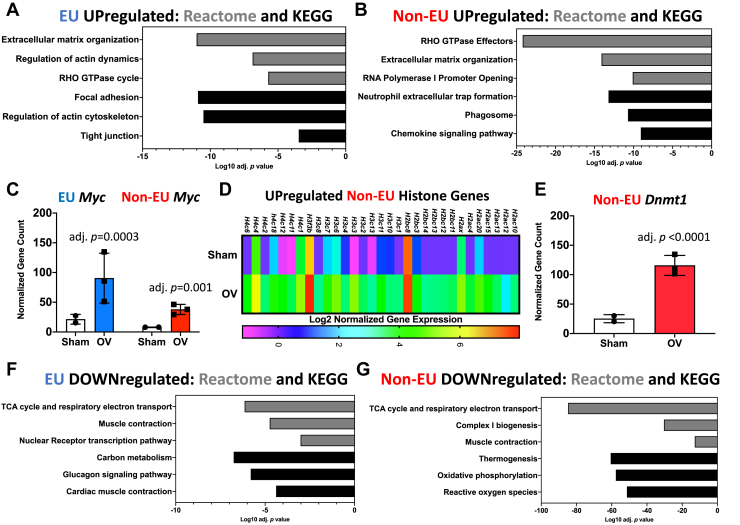
***Experiments 2 and 3*: RNA-sequencing of EU- and Non-EU–labeled mRNA from plantaris after 72 h of mechanical overload.***A*, pathway analysis of genes upregulated in the EU-labeled fraction in OV *versus* sham. *B*, pathway analysis of genes upregulated in the Non-EU–labeled fraction in OV *versus* sham. *C*, *Myc* mRNA levels in the EU and Non-EU fractions. *D*, histone genes elevated in the Non-EU fraction during overload. *E*, *Dnmt1* mRNA levels in EU and Non-EU fractions. *F*, pathway analysis of genes downregulated in the EU-labeled fraction in OV *versus* sham. *G*, pathway analysis of genes downregulated in the Non-EU–labeled fraction in OV *versus* sham. EU, 5-Ethenyl uridine; OV, overload.

Most transcription in skeletal muscle is rRNA (46), and rRNA levels are further augmented specifically in myonuclei during overload (25). We conducted mRNA profiling to understand global transcriptional dynamics in the non-rRNA pool during growth using 5-Ethenyl uridine (EU) metabolic labeling (25) (Tables S4–S9). In the EU (nascent, *Experiment 2*) and non-EU (not actively transcribed and presumably stable, *Experiment 3*) mRNA fractions, ECM remodeling was among the most upregulated processes during the last 5 h of overload *versus* sham (Fig. 2, *A* and *B*) (Tables S4–S6, and S8). *Myc* was also significantly higher in nascent and stable fractions relative to sham (Fig. 2*C*). Active *Myc* transcription during overload could be facilitated by hypomethylation of its promoter in myonuclei (19). Thus, ECM and *Myc* gene expression are highly regulated during the acute phase of muscle loading. In addition, RHO-GTPase genes were higher in both fractions with overload relative to sham (Fig. 2, *A* and *B*). RHO-GTPases are implicated in muscle mass regulation, but their role in load-induced hypertrophy is still being defined (47). Genes related to epigenetic control of gene expression (specifically histones and *Dnmt1*) were enriched in the non-EU fraction with overload (Fig. 2, *D* and *E*). Myonuclear histone turnover (48) and dynamic regulation of DNA methylation in myonuclei (19, 23, 49) likely facilitates hypertrophic gene expression and adaptation in muscle fibers.

In the EU and non-EU fractions, oxidative metabolism-related gene expression was lower during overload (Fig. 2, *F* and *G*) (Tables S7 and S9); these data inform the findings from *Experiment 1*. *Pgc1α* (*Ppargc1a*), a core regulator of mitochondrial biogenesis (50, 51), was among genes that were lower in both fractions during overload (Tables S4 and S5). Apart from epigenetic-related genes, most mRNA differences between sham and overload at the pathway level were attributable to differences in both nascent transcription and, presumably, enhanced mRNA stability.

### Experiment 4: The myonuclear transcriptome after 72 h of mechanical overload

**Figure 3:**
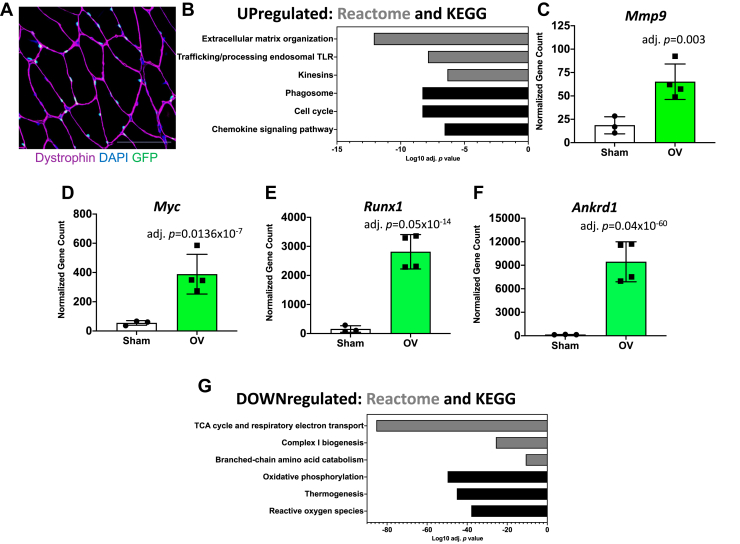
***Experiment 4*: RNA-sequencing of myonuclear RNA from plantaris muscle after 72 h of mechanical overload.***A*, image showing myonuclear GFP labeling, DNA (DAPI), and dystrophin *via* histochemistry in a doxycycline-treated HSA-*GFP* mouse (see refs. (19) and (99)); the scale bar represents 100 μm. *B*, pathway analysis of upregulated genes specifically in FANS-isolated myonuclei in OV *versus* sham. *C*, myonuclear *Mmp9* mRNA levels in sham and overload determined by RNA-seq. *D*, *Myc* mRNA levels in sham and overload determined by RNA-seq. *E*, *Runx1* mRNA levels in sham and overload determined by RNA-seq. *F*, *Ankrd1* (*Marp1*) mRNA levels in sham and overload determined by RNA-seq. *G*, pathway analysis of downregulated genes specifically in FANS-isolated myonuclei in OV *versus* sham. FANS, fluorescent activated nuclear-sorted; OV, overload.

To understand what genes were specifically regulated in muscle fibers during rapid muscle growth, we conducted RNA-seq on fluorescent activated nuclear-sorted (FANS)-purified myonuclei using the HSA-*GFP* mouse (Fig. 3*A*) (19, 52). Relative to sham, genes related to ECM remodeling (primarily collagens, matrix metalloproteinases, and secreted factors) and immune signaling (namely chemokines) were most highly enriched in myonuclei from overloaded muscle (Fig. 3*B*) (Tables S10 and S11). We and others have reported that matrix metalloproteinase 9 (*Mmp9*) is responsive to loading in skeletal muscle and is a key component of muscle growth (53, 54, 55). We confirm here that *Mmp9* is enriched in myonuclei during overload *in vivo* (Fig. 3*C*). Interstitial cells of the muscle microenvironment are generally viewed as the primary contributors to ECM deposition and turnover. Emerging evidence suggests that muscle fibers also play a major role in ECM remodeling (53, 56, 57, 58), which the current data reinforces.

Numerous cell cycle regulators were enriched in myonuclei after overload (Fig. 3*B*) which included *Myc* (top 30 upregulated gene, adj. *p* = 0.0136 × 10^−7^) (Fig. 3*C*) (Table S11). *Runx1*, another transcription factor, was highly abundant and enriched in myonuclei by overload (Log2FC = 4.1, adj. *p* = 0.05 × 10^−14^) (Fig. 3*E*); it was also markedly higher in total RNA, EU, and non-EU fractions (Tables S1, S4 and S5). *Runx1* induction during overload is intuitive since it regulates muscle mass, myofibrillar organization, and autophagy in myofibers (59). RUNX1 is also known to interact and complex with MYC (60, 61). *Ankrd1* (*Marp1*, also *Carp1*) was the most upregulated gene with overload in myonuclei (Log2FC = 6.0, adj. *p* = 0.0127 × 10^−62^) as well as the EU fraction (Log2FC = 5.1, adj. *p* = 0.038 × 10^−60^) (Fig. 3*F*) (Tables S4, S5, and S10); however, it was only the 1495th most differentially regulated gene in the total RNA dataset (Table S1). *Ankrd10* was also among the most upregulated genes in myonuclei and the EU fraction. *Ankrd1* localizes in myotendinous junction myonuclei (62) and is induced by eccentric exercise in rodent and human muscle (63, 64). Perhaps *Ankrd1* upregulation during mechanical overload is partially explained by muscle lengthening and/or myotendinous junction remodeling (35, 65). These results highlight the power of EU-labeling and myonucleus-specific transcriptomics for identifying potentially important genes for muscle growth. Our findings also provide impetus for further investigation of *Ankrd1* during muscle hypertrophy. The category of genes most downregulated during overload in myonuclei was oxidative metabolism (Fig. 3*G*) (Table S12). Thus, the total mRNA and EU results are likely driven by changes within the myofiber.

### Experiment 5: The Myc-controlled transcriptome in plantaris muscle fibers

**Figure 4:**
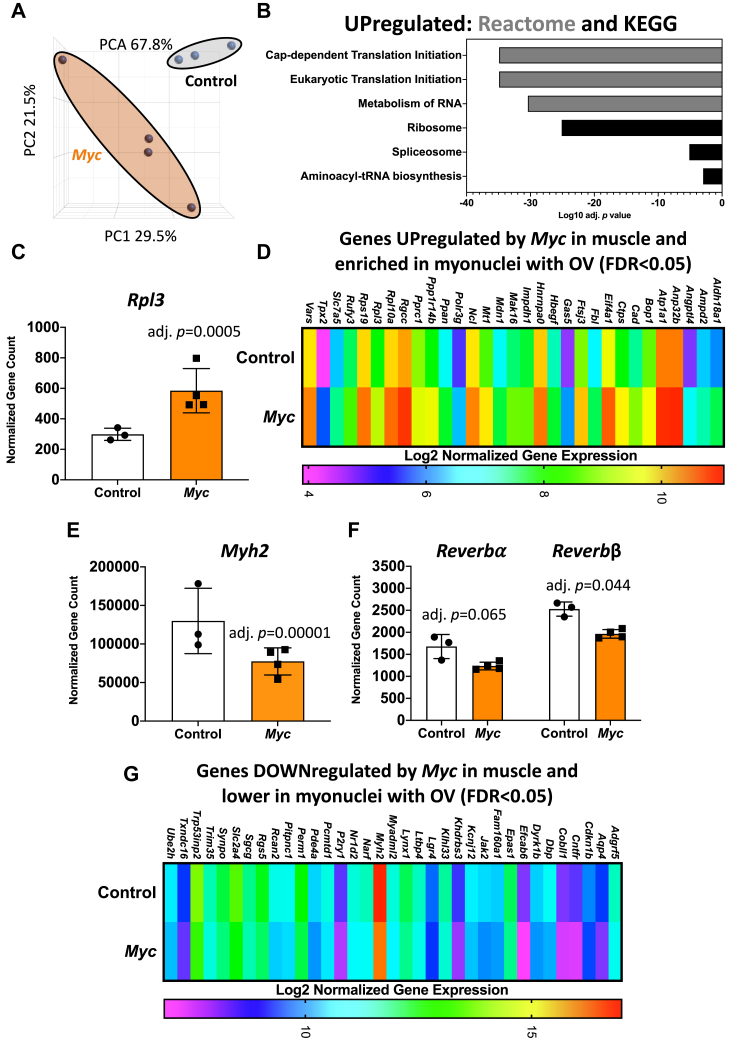
***Experiment 5*: RNA-sequencing of total RNA from plantaris muscle of HSA-*Myc* mice following a single pulse of *Myc*.***A*, PCA plots from doxycycline-treated HSA-*Myc versus* littermate HSA-*rtTA* (Control) mice (generated using DESeq2 normalized gene counts). *B*, pathway analysis of upregulated genes after *Myc* overexpression. *C*, *Rpl3* mRNA levels after *Myc* overexpression. *D*, genes upregulated by *Myc* in muscle and also enriched in myonuclei during 72 h of overload. *E*, *Myh2* mRNA levels after *Myc* overexpression. *F*, *Reverbα* (*Nr1d1*) and *Reverbβ* (*Nr1d2*) after *Myc* overexpression. *G*, genes upregulated by *Myc* in muscle also enriched in myonuclei during 72 h of overload. OV, overload; PCA, principal component analysis.

We generated a doxycycline-inducible HSA-*Myc* mouse to experimentally define the MYC regulatory network in plantaris muscle fibers. A pulse of *Myc* was driven *via* doxycycline in water overnight followed by a 12-h period without doxycycline. Principal component analysis revealed stark differences between control and *Myc* overexpression (Fig. 4*A*). At the pathway level, ribosome biogenesis-related genes such as ribosomal proteins and eukaryotic initiation factors were most upregulated relative to controls (Fig. 4*B*) (Table S13). A MYC pulse induced gene expression of the large ribosomal subunit protein *Rpl3* (Log2FC = 1.95, adj. *p* = 0.00053) (Fig. 4*C*). *Rpl3* was also higher in myonuclei during overload, and its muscle-specific paralog *Rpl3l* was lower (adj. *p* < 0.05) (Table S10). Upregulation of *Rpl3* has been implicated in robust hypertrophy in mouse (26) and human muscle (29). *Rpl3* may influence growth *via* ribosome specialization (66), but more work is needed in this area. The induction of *Rpl3* by overload and *Myc* alongside increased levels of the rRNA transcription-associated genes *Bop1* (67, 68), *Ftsj3* (69), *Polr3g* (70), *Rpl10a* (71), and *Rps19* (72), could also be a sign of enhanced ribosome biogenesis. In total, 31 upregulated genes were common to *Myc* overexpression in muscle fibers and myonuclei with overload (Fig. 4*D*).

Approximately, 100 genes were downregulated (adj. *p* < 0.05) by MYC in the plantaris (Table S13). MYC strongly regulates microRNA expression (8, 73, 74, 75, 76, 77). Repressed genes with MYC induction are potentially attributable to MYC-controlled microRNA-mediated mRNA destabilization and degradation. MYC may also repress gene expression *via* regulating DNA methylation and chromatin remodeling (3, 4, 12), as well as through specific protein-protein interactions (10, 11). The abundant myosin heavy chain type 2a gene *Myh2* was repressed by MYC (Log2FC = −0.75, adj. *p* = 0.013) (Fig. 4*E*), similar to what occurred in myonuclei during overload (Table S10). Type 2a myosin is associated with oxidative metabolism in murine muscle (78, 79). Lower *Myh2* may be part of a Warburg-like program that accompanies rapid muscle growth (38, 39). *Reverbα* (*Nr1d1*, Log2FC = −1.36, adj. *p* = 0.065) and *Reverbβ* (*Nr1d2*, Log2FC = −1.31, adj. *p* = 0.044) mRNA levels were lower with MYC overexpression (Fig. 4*F*) and in myonuclei with overload (Table S10). In cancer cells, MYC promotes *Reverbα* and *Reverb*β expression (80) which affects the core clock gene *Bmal1* (80, 81), circadian rhythm, and cell metabolism (80). Thus, MYC control of *Reverb*s could be unique in muscle fibers. In total, 35 downregulated genes were common to MYC overexpression in muscle fibers and myonuclei with overload (Fig. 4*G*).

To corroborate MYC regulation of target genes in muscle, we compared our overload myonuclear and *Myc* overexpression RNA-seq data to published MYC ChIP-seq data from myogenic cells (77). Of genes regulated by both overload and MYC (Fig. 4, *D* and *G*), *Anp32b*, *Aqp4*, *Atp1a1*, *Cdkn1b*, *Cntfr*, *Epas1*, *Ftsj3*, *Jak2*, *Ncl*, *Nr1d2/Reverbβ*, *P2ry1*, *Pcmtd1*, *Rpl3*, and *Slc7a5* featured MYC occupancy in myogenic cells. After differentiation, MYC-binding peaks on all these genes except *Aqp4*, *Atp1a1*, and *P2ry1* were altered, indicating regulation by MYC in dynamic conditions. Of note, MYC binding to *Ankrd1* increased during myotube formation (77). Brief *Myc* overexpression in the soleus did not induce *Ankrd1*, so MYC may function cooperatively with another factor that is induced during overload to regulate this gene (14, 82). To confirm MYC transcription factor binding of myonuclear DNA during mechanical overload, we utilized our RNA-seq data to perform epigenetic Landscape *In Silico* deletion Analysis (Lisa) (37). Lisa incorporates transcriptome input data with an extensive library of publicly available transcription factor ChIP-seq and global chromatin accessibility profiles to infer transcriptional regulators. Leveraging our MYC overexpression RNA-seq data as a control (*Experiment 5*), MYC/MYCN was the highest ranked transcription factor driving upregulated genes (Table S14), confirming the accuracy of Lisa. Using the first 500 differentially regulated genes in each direction from *Experiment 4*, MYC was in the top 5% of transcription factors controlling upregulated genes (*p* = 1.7 × 10^−24^) in myonuclei during overload and the top 10% for controlling downregulated genes (*p* = 2.4 × 10^−11^) (Table S15). Motif target prediction suggested that MYC regulates *Rpl3* in myonuclei during overload (top 45% of genes targeted by MYC) (Table S16). Ribosome biogenesis-associated *Bop1* (top 15%), *Ftsj3* (top 15%), *Polr3g* (top 1%), and *Rps19* (top 15%) had high regulatory potential by MYC during overload according to H3k27ac ChIP-seq (promoter/enhancer) information; *Ncl* was also in the top 1% (Table S17). MYC had regulatory potential for *Nr1d2/Reverbβ* during overload (top 35% of gene targets) (Table S18). All together, these data suggest that MYC controls gene expression in myonuclei during loading-induced hypertrophy.

## Discussion

**Figure 5:**
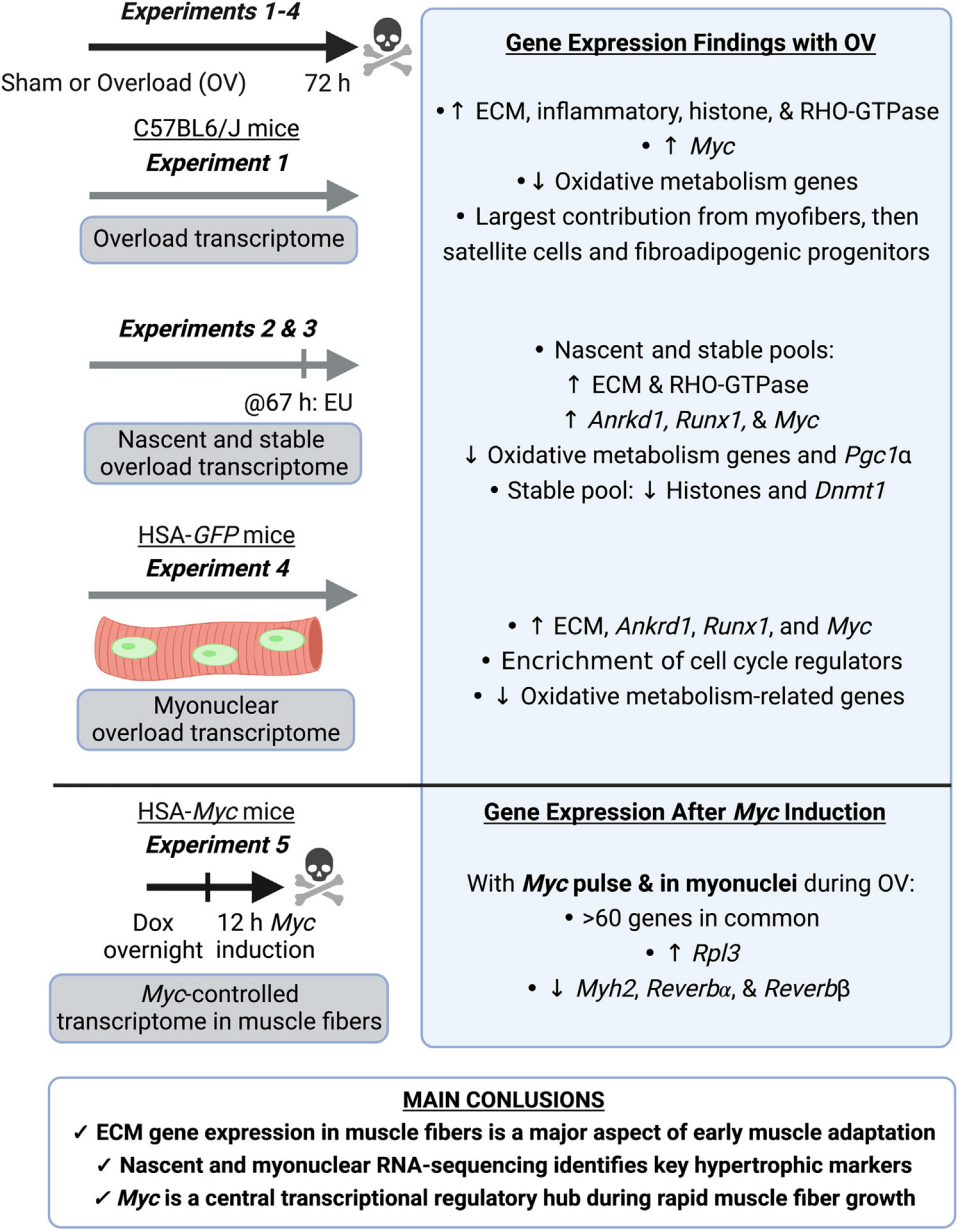
**Summary of key findings from *Experiments 1 to 5***.

Interrelated RNA-seq datasets define the early phase of growth processes in differentiated muscle fibers (Fig. 5). Lower oxidative metabolism-related gene expression during the onset of rapid muscle growth is due to changes in mRNA transcription and stability and occurs specifically in myonuclei. The overload datasets also revealed an under-appreciated role for muscle fibers in ECM remodeling during adaptation. Regulation of several collagens and remodeling enzymes such as *Mmp*s by active transcription, transcript stability, and in myonuclei emphasizes the importance of ECM dynamics for muscle hypertrophy (56). The non-EU RNA-seq data suggests elevated epigenetic-related gene expression during overload reflects greater mRNA stability; the mechanism underlying the change in mRNA stability in muscle during hypertrophy deserves further investigation. We identified key genes that are actively transcribed in muscle and enriched in myonuclei during *in vivo* muscle growth, such as *Runx1* and *Ankrd1*. RUNX1 regulates muscle mass (59) and participates in ribosome biogenesis (83), as well as interacts with MYC (60, 61). ANKRD1 associates with titin’s N2A element, a major mechanosensory and signaling hub in skeletal muscle (84, 85). It also locks titin to the thin filament, regulates passive force, and protects the sarcomere from mechanical damage (86). Some evidence suggests ANKRD1 inhibits TNF*α*-induced NFκB signaling (87) and affects androgen receptor signaling (88) in myogenic cell culture. Given these functions, ANKRD1 may be critical for successful muscle hypertrophy. *Ankrd1* was not among the most differentially expressed genes in the total RNA dataset but emerged in the nascent and myonuclear RNA-seq as the most highly responsive gene to mechanical overload. This mismatch highlights the utility of evaluating transcriptional dynamics and myonuclear-specific gene expression for understanding muscle adaptation. Furthermore, *Runx1* and *Ankrd1* are typically upregulated after muscle denervation (59, 87), suggesting a compensatory response to counteract atrophy in that condition.

MYC protein localizes in myonuclei during loading-induced skeletal muscle growth (15, 27). Genetic *Myc* induction recapitulates diverse aspects of the loading response in muscle fibers. These changes include downregulation of *Reverbα*, *Reverbβ*, and *Myh2*, along with increased *Rpl3*. Published ChIP-seq data in myogenic cells (77) as well as *in silico* transcriptional regulator analysis (37) using our myonuclear RNA-sequencing data corroborates the association of MYC with *Reverb*β and *Rpl3*, along with numerous other genes. The ChIP-seq also revealed a potential interaction with *Ankrd1* (77) that did not emerge in our muscle-specific MYC overexpression experiment. Lower levels of *Reverb*s during overload could have implications for circadian regulation and metabolism in muscle fibers (80); this is salient since MYC is exercise-responsive and exercise shifts the circadian rhythm in skeletal muscle (89, 90, 91, 92). Perhaps an exercise-mediated shift in muscle circadian rhythm is controlled by MYC, but more work is needed in this area, especially with respect to resistance exercise. Altered *Rpl3* by mechanical overload and MYC expression could facilitate muscle-specific growth *via* ribosome specialization (66, 93). MYC is also known to be a potent driver of ribosome biogenesis in muscle (21, 22). We previously reported that ribosome biogenesis increases in total and nascent RNA pools following 72 h of synergist ablation in the mouse (20, 23, 25, 94). Thus, MYC appears central to the regulation of rRNA synthesis and ribosome assembly, processes hypothesized to be necessary for sustained hypertrophy in response to loading (95, 96, 97). MYH2 protein and oxidative fiber proportion increases after prolonged muscle overload (98). MYC-mediated and early *Myh2* downregulation during overload may relate to an acute glycolytic preference during rapid hypertrophy (38).

The induction of MYC in instances of attenuated muscle plasticity such as aging, where MYC activity may be blunted during hypertrophy (15), could restore adaptive potential and increase muscle mass. Future experiments involving simultaneous muscle-specific inducible knockout of *c-Myc* and its several analogous family members (99), myonuclear MYC ChIP-seq, and MYC protein–protein interactome analysis during overload will provide more granular insight on the MYC regulatory network during muscle hypertrophy. Since differentiated myofibers can sustain high levels of oncogene expression without tumor formation, we suggest that MYC in muscle fibers induced by loading is a core component of rapid yet functional adaptive remodeling. Collectively, our data are a rich resource for understanding transcriptional dynamics and MYC regulation during the onset of loading-induced muscle fiber growth.

## Experimental procedures

### Animals and animal procedures

All animal procedures were approved by the University of Kentucky and the University of Arkansas IACUC. Mice were housed in a temperature and humidity-controlled room, maintained on a 14:10-h light-dark cycle, and food and water were provided *ad libitum* throughout experimentation. Animals were sacrificed *via* a lethal dosage of sodium pentobarbital injected intraperitoneally or CO_2_ asphyxiation followed by cervical dislocation.

Female C57BL6/J mice were obtained from the Jackson Laboratory for *Experiments 1 to 3*. Female HSA-rtTA^+/−^-;TRE-H2B-GFP^+/−^ (HSA-*GFP*) mice were generated as previously described by us (19, 52) for *Experiment 4*. The TRE-H2B-GFP mouse was originally obtained from the Jackson Laboratory (005104, bred to homozygosity by our laboratory) (100). For *Experiment 5*, male HSA-rtTA^+/−^-;TRE-Myc^+/−^ (HSA-*Myc*) were generated by crossing homozygous HSA-*rtTA* mice (52) with heterozygous TRE-*Myc* mice (101); HSA-*rtTA* littermate mice were used as controls. All mice were genotyped as described (52, 102). HSA-*GFP* mice were treated with low-dose doxycycline (0.5 mg/ml doxycycline in drinking water with 2% sucrose) for 5 days to label myonuclei with GFP. HSA-*Myc* and littermate control mice were treated with doxycycline water overnight, and this water was replaced with unsupplemented water for 12 h prior to being euthanized. All mice were at least 2 months of age at the time of experimentation.

For *Experiments 1 to 4*, synergist ablation overload of the plantaris was performed as described (19, 34). Synergist ablation involves removal of ∼50% of the gastrocnemius–soleus complex while under anesthesia, followed by ambulatory cage behavior for 72 h. Sham surgery (control condition) involved all the steps of synergist ablation but without removal of muscle. For *Experiments 1 to 3*, mice were injected with EU 5 h prior to being euthanized (25). Briefly, mice were given an intraperitoneal injection of 2 mg of EU (Jena Biosciences) suspended in sterile PBS. For *Experiment 4*, myonuclei were isolated *via* FANS (19). Plantaris muscles were harvested immediately after being euthanized. Muscle was minced and homogenized *via* Dounce in a sucrose buffer with RNAse inhibitors. After straining through 40 μm filters, the nuclear suspension was pulsed with propidium iodide to label DNA. GFP+/PI+ myonuclei were sorted using FANS into TRIzol LS for RNA isolation.

### RNA isolation and sequencing

For *Experiments 1*, *2*, *3*, and *5*, plantaris RNA was isolated using TRI Reagent (Sigma-Aldrich). Tissue was homogenized using beads and the Bullet Blender Tissue Homogenizer (Next Advance) or the Fisher Bead Mill (Fisher). Following homogenization, RNA was isolated *via* phase separation by addition of bromochloropropane or chloroform and then by centrifugation. The aqueous phase was transferred to a new tube and further processed on columns using the Direct-zol Kit (Zymo Research) (23). For *Experiment 4*, RNA was isolated by Norgen. *Experiments 1*, *4*, and *5* utilized Poly-A enrichment. For *Experiments 2* and *3*, RNA was depleted of rRNA using the NEBNext rRNA Depletion Kit (New England Biolabs) prior to isolation of EU- and non-EU–labeled RNA. A total of 4 × 1 μg RNA reactions were used per sample for rRNA depletion and pooled for the EU pulldown. The EU- and non-EU–labeled RNA fractions were isolated using the Click-iT Nascent RNA Capture kit (ThermoFisher) per the manufacturers protocol. cDNA libraries were constructed using NEBNext Ultra# II RNA Library Prep kit with NEBNext Multiplex Oligos for Illumina (New England Biolabs). EU pulldowns were unsuccessful for one sham experiment, so sequencing for that group was n = 2. Library preparation for *Experiment 4* was low input and utilized the SMARTer pico kit (TaKaRa), as described by the National Genomics Infrastructure at SciLifeLab. RNA for *Experiments 1*, *2*, *3*, and *5* were sequenced by Novogene on an Illumina HiSeq using 150 bp paired-end sequencing, as we have done previously (79, 103). *Experiment 4* was sequenced by the SciLifeLab on a NovaSeq 6000 (150 bp paired-end).

### Transcriptomic analyses

Raw counts from RNA-seq were used as inputs into R (Version 4.1.0) or Partek Flow. Alignment was performed using STAR with mmu39. After filtering low-expressed genes, *DESeq2* (Version 1.34.0) was used for normalization and differential analyses of RNA-seq data to identify differentially expressed genes (DEGs) with pairwise comparisons (104). DEGs were identified with a false discovery rate (Benjamini-Hochberg method) adjusted *p*-value <0.05. DEGs with a log2 fold change (Log2FC) over 1 and adj. *p* < 0.05 were used for downstream functional analysis in *Experiments 1 to 4*. A fold-change cut-off was not used for *Experiment 5*. Kyoto Encyclopedia of Genes and Genomes and Reactome (https://reactome.org/) were utilized for pathway analysis. We utilized clusterProfiler (Version 4.4.1), ReactomePA, ggplot2 (3.15), and ConcensusPathDB (105) with mouse as the reference organism.

### Digital cell sorting with CIBERSORTx

CIBERSORTx (https://cibersortx.stanford.edu/) is a machine learning method that enables prediction of cell type proportions from bulk tissue analysis using single cell RNA-seq data (42). We used skeletal muscle single-cell data from 10 days muscle regeneration data from Oprescu *et al.* (43). The datasets (10X Genomics) were reanalyzed with Seurat, and cell clusters were identified with a resolution of 0.8 (106). Normalized gene expression matrices of individual cells were used to create a signature matrix of all cell types using default settings, and cell proportions were predicted by CIBERSORTx with 1000 permutations.

### Transcriptional regulator analysis using Lisa

Lisa was run according to recommended procedures (37). In brief, DEG lists (adj. *p* < 0.05) from *Experiments 4* and *5* were input into the online graphical user interface. Output files were downloaded and the strength of MYC regulation was determined by ranking of regulatory potential in H3k27ac ChIP-seq files. The Cauchy combination *p*-value test was used to determine overall influence of MYC.

## Data availability

Raw data are available in Gene Expression Omnibus GEO213406, and all processed data are provided in Supporting information and online webtools.

## Supporting information

This article contains supporting information.

## Conflict of interest

Y. W. is the founder of MyoAnalytics LLC. The authors have no other conflicts to declare.
